# Influence of physicochemical properties on the production of alternative healthy gummy jelly from tilapia (*Oreochromis niloticus*) skin with added Thai rice powder

**DOI:** 10.1016/j.fochx.2022.100365

**Published:** 2022-06-17

**Authors:** Sukhuntha Osiriphun, Pornchai Rachtanapun, Sutee Wangtueai, Wachira Jirarattanarangsri

**Affiliations:** a Division of Food Science and Technology, Agro-Industry, Chiang Mai University, Chiang Mai 50100, Thailand; b Cluster of Innovative Food and Agro-Industry, Chiang Mai University, Chiang Mai 50100, Thailand; c Division of Packaging Technology, Agro-Industry, Chiang Mai University, Chiang Mai, 50100, Thailand; d Cluster of Agro Bio-Circular-Green Industry (Agro BCG), Chiang Mai University, Chiang Mai 50100, Thailand; e College of Maritime Studies and Management, Chiang Mai University, Samut Sakhon 74000, Thailand

**Keywords:** Fish skin, Gelatine, Pasting properties, Rice, Sports food

## Abstract

We developed a new healthy gummy jelly from tilapia skin-derived gelatine and four rice cultivar powders. The gelatine from the tilapia fish skin was obtained using acid and alkaline extraction. The extracted gelatine comprised 96.77 ± 0.96 % protein and featured a breaking force of 5.21 ± 0.58 g, which indicates high gel strength. The fish gelatine was combined with the rice varieties to produce a gummy jelly with an enhanced nutritional value, and its flavor profile was determined. Gummy jelly products with added black sticky rice powder exhibited the highest protein and carbohydrate contents. The gummy jelly supplemented with rice berry had the highest score, with 90 % acceptance, on a nine-point hedonic scale. Microbiological analysis showed that no microorganisms were detected in any of the samples. These results indicate that fish gelatine and rice powder may be used as nutritive ingredients in sports food to improve athletic performances.

## Highlights

• Novel gummy jelly energy snack for athletes from tilapia gelatine and rice powder.
• Gelatin extracted by acid and alkaline hydrolysis from Nile Tilapia fish skin.
• Extracted gelatine is protein-rich with high gel strength.
• Tilapia gelatine and rice powder gummy jelly is rich in protein and carbohydrates.
• Potential as a healthy supplement in the food industry.

## Introduction

As food and exercise are understood to be major factors that affect personal health and well-being, consumers have become increasingly health-conscious (Tharnpichet, Jirarattanarangsri, Osiriphun, Peepathum, & Mitranun, 2019). There is now a heightened demand from health-conscious consumers for the natural health products used by athletes (Too et al., 2012). As a result, several sports drink products, such as sports powders and energy sports gels, which are portable and designated to fit with their function as natural health products, have been developed as alternatives for athletes who want to exercise longer without experiencing fatigue (Tharnpichet et al., 2019). Although many sports food supplements are available on the market, no gummy jelly products produced from fish skin waste (a protein source) and rice powder (a carbohydrate source) have yet been developed. Gummy products comprise > 50 % of the candy market in Thailand (Sudpanya, Asawaruangpipop, & Suwunnamek, 2018). A gummy jelly is a mixture of gelatine, sugar, and glucose syrup (Marfil, Anhê, & Telis, 2012), where gelatine performs the gelling function by forming a three-dimensional network (i.e., a junction zone) in the gel structure (Guo et al., 2003, Wangtueai et al., 2010). Gummy jellies can be easily consumed and are easy to transport, and in addition to being attractive to athletes for energy intake during prolonged exercise (Jiamjariyatam, 2018), they have the potential to attract the attention of young adults due to its snack-like properties and desirable health benefits (Kitpot et al., 2020).

Nile tilapia is the most economically important species of freshwater fish in Thailand. It has white flesh and a delicate flavor that is popular in terms of domestic consumption, and approximately 38 % of stocks are exported as frozen and chilled products (Ghaly et al., 2013, Wangtueai et al., 2020, Wangtueai and Vichasilp, 2015). According to data collected in 2016, the aquaculture industry yielded approximately 177,000 tons of farm-produced tilapia, of which, >8,000 tons (worth approximately 18 million USD) were exported. This large-scale production creates excess waste containing skin, scales, fins, and bones, which constitutes > 50–70 % of the raw fish material depending on level of processing and type of fish (Ghaly et al., 2013, Thai Residents Team, 2017). Fish protein is easily digested and comprises ≥ 85 % protein or amino acids, and so is a good protein source for human consumption (Abraha et al., 2018). In addition, gelatine is a protein that is derived from the collagenous connective tissue, skin, and bones of animals, and is widely used in the food, cosmetics, and pharmaceutical industries (Firdayanti & Suprayitno, 2019). Gelatine can be produced from fish by-products, such as the skin, bone, and scales, which contain various amino acids, including high levels of glycine, proline, and hydroxyproline. The properties of fish gelatine are influenced by its molecular weight, structural fragments, and amino acid composition, wherein the imino acid groups (i.e., proline and hydroxyproline) are of particular importance (Firdayanti and Suprayitno, 2019, Wangtueai and Noomhorm, 2009). Furthermore, the extraction method, fish type, and treatment intensity all determine the potential uses of the produced gelatine. In general, glycine, proline/hydroxyproline, and alanine are the predominant amino acids, with the gelatin containing these components in percentages of 33, 20, and 11 %, respectively (Firdayanti & Suprayitno, 2019). Furthermore, fish gelatine clouds may be used to produce the highly functional fish gelatine hydrolysate using enzyme hydrolysis, and the application of fish gelatine hydrolysate to food products could enhance the quantity of bioactive peptides and improve the nutritional properties (Wangtueai, Phimolsiripol, Vichasilp, Regenstein, & Schöenlechner, 2020). Furthermore, the use of gelatine hydrolysate as a dietary protein source to support connective tissue protein remodeling after exercise has previously been investigated (Holwerda & van Loon, 2021).

In Thailand, rice is a staple food that is a nutritional source of carbohydrates, and so may be used as a supplementary energy product for athletes (Tharnpichet et al., 2019). Different combinations of rice powders may be prepared that comprise a mixture of carbohydrate sources, and these allow for greater absorption and utilization, which can be beneficial during prolonged exercise (Too et al., 2012).

In this study, we employed gelatine extracted from fish skin waste to develop a gummy jelly that is supplemented with different types of rice powders, namely Thai jasmine rice (Khaw Dok Mali 105 cultivar), Thai white rice (Sao Hai cultivar), black sticky rice (Kam cultivar), and rice berry (Thai colored indica rice cultivar rice berry), to study the influences of the various physicochemical parameters on the quality of products that could potentially be used as health supplements in the food industry. The hypothesis on which this study is based is that tilapia skin waste could be successfully used in combination with rice flours to form nutritionally appropriate gummy jelly energy snacks.

## Materials and methods

### Materials

Fresh tilapia skin was kindly supplied by the fish industry of the Nakorn Phanom province, Thailand. The skin samples were transported to the laboratory under ice to maintain the temperature at 4 °C, and were stored between − 18 and − 20 °C prior to use. Before use, the fresh tilapia skin was thawed by storing it in a refrigerator at 4 °C for approximately 24 h. After washing by rinsing with tap water, it was cut into pieces (2.0 × 2.0 cm^2^) and washed again with water (Jongjareonrak, Benjakul, Visessanguan, & Tanaka, 2006). The four rice varieties, namely Thai jasmine rice (Khaw Dok Mali 105 cultivar), Thai white rice (Sao Hai cultivar), black sticky rice (Kam cultivar), and rice berry (Thai colored indica rice cultivar rice berry), were purchased from B Natural Co., ltd., and Chiang Mai, Thailand. All chemicals used in this study were purchased from Union Science Co., ltd. (Chiang Mai, Thailand) with 95 % purity.

#### Chemical composition

The thawed fresh tilapia skin was pretreated with 95 % ethanol at room temperature (approximately 30 °C) for 1 h to remove any lipids. Using the methods of the Association of Official Agricultural Chemists (AOAC, 2000), the moisture (AOAC method No. 950.46), ash (method No. 920.153), protein (AOAC method No. 928.08), and fat contents (AOAC method No. 963.15) of the fresh tilapia skin and rice powder samples were determined. The nitrogen content was then quantified using nitrogen combustion experiments, and the protein content was calculated by multiplying the nitrogen content by the nitrogen conversion factor for fish skin (6.25) and rice powder (5.95).

#### Analysis of the pasting properties

The pasting properties of all rice flour samples were characterized using a Rapid Visco Analyzer (RVA) (RVA-4 D, Newport Scientific, New South Wales, Australia) according to the American Association of Cereal Chemists (AACC) method No. 61–2 (AAAC, 2000). A rice powder sample with a moisture content of 14 % was passed through a 120-mesh sieve, weighed to 3.000 ± 0.005 g, placed in an aluminum cup filled with water (25 ± 0.005 g, 11 % concentration), mixed with a plastic mixing blade, and placed in the Rapid Visco Analyzer using standard program no. 1. The samples were maintained at 50 °C for 0–1 min, heated (12.2 °C/min) to 95 °Cover 2.5 min, cooled to 50 °C (12.2 °C/min), and maintained at 50 °C for 2.1 min. The agitation speed of the RVA paddle was maintained constant at 160 rpm throughout the experiment. The pasting temperature, peak viscosity (PV), trough viscosity (TV), and final viscosity (FV) were recorded; breakdown (BD) = PV − trough viscosity; setback from trough = FV − trough viscosity (cP); setback from peak = FV − PV (cP), breakdown is the difference between the highest viscosity achieved during heating at 95 °C and the lowest viscosity achieved during heating at 95 °C, setback from through is difference between the paste viscosity upon cooling at 50 °C and the lowest viscosity achieved during heating at 95 °C, and setback from peak is starch retrogradation tendency after gelatinization and cooling.

#### Chemical extraction of gelatine from the fish skin

Tilapia fish skin gelatine was obtained using the methods of Jongjareonrak et al., 2006, Sukkwai, 2012, and Firdayanti and Suprayitno (2019) with modification of the fish species employed. Initially, a sample (300 g) of the fish skin was washed under running tap water for approximately 1 h to remove any superfluous materials. The cleaned fish skin was then pretreated with 0.1 mol/L NaOH for 2 h at 25 °C to eliminate any unwanted proteins, wherein a 1:3 ratio of skin/solution (w/v) was employed. The alkaline-treated skin was then soaked in 0.5 mol/L acetic acid for 2 h at 55 °C to loosen the natural conformation of the collagen protein and facilitate dissolution (Jongjareonrak et al., 2006). After a given treatment time (2 h), the treated fish skin was neutralized using running tap water, followed by blending with distilled water (1:1, w/v) three times, and extracted at 70 °C for 5 h in a water bath. The extracted gelatine was filtered through a double-layered white cotton cloth and dried at 60 °C in a hot air oven for 48 h. Subsequently, the resulting gelatine sheets were crushed into a powder particle sizes to 0.5 mm of standard mesh (30 mesh) (Firdayanti & Suprayitno, 2019), and the extraction yield and gel strength of the gummy jelly samples were determined.

#### Analysis of the gelatine yield

The gelatine yield was determined using Equation 1.

(1) Gelatine yield (%) = 100 % × weight of dry gelatine (g)/weight of initial dry fish skin

The weight of the dry fish skin waste was calculated by subtracting the moisture content determined according to AOAC (2000) from the initial wet weight (Firdayanti & Suprayitno, 2019).

#### Breaking force and deformation of the gel

The breaking force (gel strength) and deformation (elasticity/deformability) of the gel obtained from tilapia fish gelatine were determined using the method described by Benjakul, Visessanguan, and Srivilai (2001). The gels were equilibrated at room temperature (28–30 °C) for 1 h before analysis. Five cylindrical gel samples (2.5 cm diameter) were cut into pieces of 2.5 cm in length. The textural properties of the gels were determined using a texture analyzer (Model TA-XT2, Stable MicroSystems, Surrey, UK) equipped with a 5 mm-diameter spherical plunger probe (P/0.5R). A penetration test was performed at a constant depression speed of 60 mm/min on the cut surface of the gel sample until it was punctured. The force required to puncture the gel (breaking force, g) and the distance at which the probe entered into the gel (deformation, mm) were recorded.

### Product development of gummy jelly with added rice powder

To prepare the gummy jelly samples, the gelatine powder from fish skin (5 g) was dissolved in water (50 g, w/v), cane sugar (80 g) was added, and the solution was heated at 100 °C for 5 min. Subsequently, rice powder (10 g per 100 g serving, 1:2 gelatine:rice powder ratio) was added to the dissolved gelatine solution, and the mixture was poured into molds. The resulting gummy jelly was obtained following refrigeration for 10–20 min. All products were stored in a vacuum-sealed container at room temperature (25 ± 2 °C) prior to use. The method of Kongwan, Tipkanon, and Chusuk (2017) was employed with some modification of the rice varieties employed. It should be noted here that for consumer acceptance, the optimal formulation of a jelly product from germinated brown rice powder (GBRP) should consist of 10–12.5 % GBRP, 5–5.83 % gelatine powder, and 82.5–85.0 % water. The gummy jelly with added rice powder follows the Thai Food and Drug Administration (Thai FDA) regulations outlined in the Good Manufacturing Practice (GMP) guidelines for jam, jelly, and marmalade in hermetically sealed containers, standard number 213 (Thai FDA, 2000), and the Thai community product standard of soft jelly TCPS Number 519/2547 of the Thai Industrial Standards Institute (2004).

#### Physical, chemical, and microbiological analyses

According to the AOAC method (2000), the physicochemical analysis of the gummy jelly with added rice powder was conducted in terms of its protein and carbohydrate contents. The color values (L*, a*, b*) of all samples were measured using a Minolta colorimeter (Konica Minolta, CR-400 Series, Japan) Park, 2005, Food and Agriculture Organization, 2001. The total color difference (ΔE*) of the gel was calculated using Equation 2 (Gennadios, Weller, Hanna, & Froning, 1996):

ΔE* = $\sqrt{{{(\Delta L}^{*})}^{2}+{{(\Delta a}^{*})}^{2}+{{(\Delta b}^{*})}^{2}}$ (eq. 2).where ΔL*, Δa*, and Δb* are the differences between the corresponding color parameters of the sample and that of the white standard (L* = 93.6, a* = −0.94, and b* = 0.40).

#### Determination of gel strength

The gummy jelly samples containing the added rice powder were prepared for analysis of the gel strength using the method described by Kaewdang, Benjakul, Prodpran, Kaewmanee, and Kishimura (2015) with some modifications. More specifically, the gummy jelly contained added rice powder (1:2 ratio of jelly to rice powder) from each of the rice varieties in addition to other raw ingredients (i.e., cane sugar and water) as per the method of Kongwan et al. (2017). A 6.67 % (w/v) gelatine solution was prepared in the following manner: the gelatine solution was dissolved in distilled water, heated at 60 °C, stirred until there was complete dissolution, and then transferred to a cylindrical mold with a diameter of 3 cm and a height of 2.5 cm. Prior to further analysis, the gel was set by cooling at 4 °C for 18 h, and the gel strength was determined while the sample was still in the temperature range of 8–10 °C using a texture analyzer with a load cell of 5 kg and a crosshead speed of 1 mm/s with a control of speed. A 1.27 cm-diameter (P/0.5R) flat-faced cylindrical Teflon plunger was used to measure the ability of a colloidal dispersion to develop and retain a gel form. The maximum force (g) required for the plunger to penetrate 4 mm into the gelatine gels was recorded.

#### Microbiological analyses

The total plate count (TPC), yeast count, and mold count for all treatments of the gummy jelly samples were determined according to the AOAC method (2000).

### Sensory analysis

To understand the authentic taste attributes and perceived sensory characteristics of the prototype gummy jelly products, descriptive sensory analysis and consumer acceptability tests were conducted for four different types of sample using a nine-point hedonic scale (Kim, Hong, Song, Shin, & Kim, 2010). The test subjects consisted of fifty panelists between 18 and 25 years old. For the descriptive analysis, the terms relevant to the description of the product quality were “color” for the color attribute, “fishy odor” for the aroma attribute, “fish flavor” and “flavor” for the flavor attribute, “sweetness” for the taste attribute, and “viscosity” for the texture attribute (Moreira, Carvalho, Santos, Oliveira, & Costa, 2018). The fifty panelists, who served as judges for the sensory evaluation, were habitual consumers of gummy jelly and protein drink products. The judges were requested to record their degree of preference for the main taste attributes of the proposed gummy jelly products: color, fishy odor, fish flavor, flavor, sweetness, viscosity, and overall liking, according to the nine-point hedonic scale. In addition to the sensory evaluation, another nine-point “just about right” hedonic scale that ranged from 1 (dislike extremely) to 9 (extremely like) was used. The “just about right” scale ranged from “too-weak flavor” to “too-strong flavor.” The proposed gummy jelly products were also evaluated by the fifty panelists in terms of product acceptance and the likelihood of purchase in the future. All samples were served in paper cups in a randomized order and identified with three random digits (Viriyajaree, 1992).

### Statistical analysis

All measurements were performed at least three times, and the results were reported as the corresponding means ± standard deviations (SDs). The significance of differences between various treatments was evaluated by one-way analysis of variance and the least significant difference test using SPSS version 17.0 software (SPSS Inc., Chicago, IL, USA). The significance level was set to *P* ≤ 0.05.

## Results and discussion

Tilapia skin from various food industries in Nakorn Pathom province, Thailand, was prepared as described above prior to analysis.

### Chemical composition

The tilapia skin was found to contain 70.11 ± 0.85 % moisture, 29.68 ± 1.32 % protein, 12.07 ± 0.44 % fat, and 0.20 ± 0.08 % residues (ash) (Table 1), while the gelatine from the fish skin contained 5.10 ± 0.20 % moisture, 96.77 ± 0.96 % protein, 0.63 ± 0.35 % fat, and 0.60 ± 0.18 % residues (ash). These results coincide with the results of Inthuserdha and Chiradetprapai (2016), with the exception of the fat content, which was higher in their study (i.e., 12.07 ± 0.44 %).

**Table 1:** Chemical compositions of tilapia fish skin and tilapia gelatine.

Product	Composition (means ± SDs (*n* = 3))			
	Moisture (% wb)	Protein* (%)	Fat (%)	Ash (%)
Fish skin	70.11 ± 0.85	29.68 ± 5.14	12.07 ± 0.44	0.20 ± 0.08
Gelatine	5.10 ± 0.20	96.77 ± 0.96	0.63 ± 0.35	0.60 ± 0.18

* Conversion factor = 6.25.

The protein and carbohydrate contents of the rice varieties are listed in Table 2. As indicated, the Thai jasmine rice, Thai white rice, Thai black sticky rice, and rice berry samples contained 6.32 ± 0.16, 6.05 ± 0.04, 7.89 ± 0.14, and 7.40 ± 0.02 % protein, respectively, and 83.99 ± 0.54, 84.92 ± 0.63, 86.05 ± 1.02, and 82.45 ± 0.85 % carbohydrates, respectively. These results are similar to those reported by the FAO (2001) for white rice flour (5.95 % protein and 80.13 % carbohydrates) and brown rice flour (7.23 % protein and 76.48 % carbohydrates) Food and Agriculture Organization, 2001.

**Table 2:** Chemical compositions of the different varieties of rice powder.

Rice powder	Composition (means ± SDs (*n* = 3))	
	*Protein (%)	Carbohydrate (%)
Thai jasmine rice	6.32 ± 0.16	83.99 ± 0.54
Thai white rice	6.05 ± 0.04	84.92 ± 0.63
Thai black sticky rice	7.89 ± 0.14	86.05 ± 1.02
Rice berry	7.40 ± 0.02	82.45 ± 0.85

* Conversion factor = 5.95.

### Pasting properties of the various flours

The pasting temperatures, or the minimum temperatures of gelatinization, for Thai jasmine rice, rice berry rice, Thai white rice, and Thai black sticky rice were determined to be 79.75 ± 0.05, 90.80 ± 0.00, 85.34 ± 0.04, and 91.50 ± 0.00 °C, respectively (Table 3). These results are in agreement with those of Yodmanee (2008), who reported gelatinization temperatures of non-waxy pigmented rice starch in the range of 69.76–81.33 °C, while those of waxy rice were 59.36–80.85 °C.

**Table 3:** Pasting properties of the various rice powders.

Pasting properties	Parameters (means ± SDs (*n* = 3))			
	Thai jasmine rice	Rice berry	Thai white rice	Thai black sticky rice
Pasting temperature (°C)	79.75 ± 0.05^d^	90.80 ± 0.00^b^	85.34 ± 0.04^c^	91.50 ± 0.00^a^
Peak viscosity (cP)	3751 ± 74^a^	1329 ± 246^c^	1614 ± 45^b^	1297 ± 63^c^
Trough viscosity (cP)	1961 ± 12^a^	1123 ± 117^c^	1341 ± 159^b^	1144 ± 41^c^
Final viscosity (cP)	3322 ± 13^a^	2653 ± 690^b^	2441 ± 9^b^	2251 ± 20^b^
Setback (cP)	−428 ± 87^c^	1324 ± 444^a^	826 ± 36^b^	954 ± 42^ab^
Breakdown (cP)	1790 ± 87^a^	207 ± 2^b^	273 ± 114^b^	155 ± 23^b^
Peak time (min)	5.33 ± 0.03^d^	6.05 ± 0.09^b^	5.67 ± 0.08^c^	6.20 ± 0.00^a^

Different lowercase letters within a row indicate significant differences (*P* ≤ 0.05).

In our study, Thai jasmine rice exhibited the highest PV of 3751 ± 74 cP, while Thai black sticky rice exhibited the lowest value (1297 ± 63 cP). The BD values of the four rice cultivars ranged from 155 ± 23 to 1790 ± 87 cP, while the FVs of the four rice cultivars ranged from 2251 ± 20 to 3322 ± 13 cP. The BD values indicate the ease with which starch granules are broken when heated after reaching maximum swelling at the peak viscosity. Thai jasmine rice showed the highest peak of the trough viscosity (1961 ± 12 cP), while the Thai white rice showed the highest peak setback value (1324 ± 444 cP). The FV was positively correlated with the amylose content and the setback value. It should be noted here that the setback values indicate the hardness of the cooled gel paste, with a high setback value corresponding to the high retrogradation of starch (Chrastil, 1990). Amylose and amylopectin play important roles in starch swelling, wherein the amylose content is negatively correlated with swelling of the starch granules (Tester & Morrison, 1990), while viscosity, retrogradation, and syneresis properties of starch gels affect visual gel deterioration during food product development (Fredriksson et al., 2000).

### Gelatine extraction of the fish skin

The gelatine extraction process, carried out by acid treatment, is used to hydrolyze the collagen present in fish skin by breaking down the intramolecular bonds of collagen triple-helix molecules. This process dissolves the minerals in the fish bone and skin, and ultimately affects the physical characteristics and amino acid compositions of the gelatine depending on the species and type of tissue, although high levels of glycine, proline, and hydroxyproline are commonly present (Firdayanti & Suprayitno, 2019). The proximate test results for the gelatine obtained from fish skin indicate a protein content of 96.77 ± 0.96 (g/100 g) (Table 1), with the extraction yield of gelatine from tilapia fish skin being 17.33 %, which is higher than that reported by Firdayanti and Suprayitno (2019) (5.4 %). The breaking force and deformation values of gelatine from the tilapia fish skin were 5.21 ± 0.58 g and 4 ± 0.00 mm, respectively. This study suggests that the skin of tilapia may be a key source of gelatine.

### Gummy jelly with added rice powder

The results of this study indicate that gummy jelly with added rice powder can be developed as an energy product for athletes. Carbohydrates are essential as they maintain blood sugar levels during exercise, while proteins are the building blocks of muscle tissue (American Dietetic Association et al., 2009). The percentage of proteins and carbohydrates in gummy jelly samples supplemented with rice powder ranged from 8.76 ± 0.43 to 10.01 ± 0.45 % and from 55.75 ± 0.51 to 69.52 ± 1.52 %, respectively (Table 4). The protein and carbohydrate contents of gummy jelly–supplemented Thai black sticky rice significantly exceeded (*P* ≤ 0.05) those of other samples and were within the recommended intake values for consumers, based on expert recommendations and reliable research evidence, thereby indicating that the proposed product is suitable for use in the food industry (Moreira et al., 2018). The recommended daily carbohydrate intake for athletes ranges from 6 to 10 g/kg body weight, while energy sports supplements should contain at least 15.0 g of carbohydrates per serving size (ANVISA, 2010, Moreira et al., 2018). Endurance athletes are advised to ingest 1.2–1.4 g of protein per kg of body weight each day (American Dietetic Association et al., 2009), as carbohydrate-protein feedings help to prolong their endurance performance and improve athletic recovery by attenuating muscle damage following heavy endurance training (Saunders, Luden, & Herrick, 2007). Furthermore, it is recommended that carbohydrate-protein gels be consumed during and immediately following exercise (Moreira et al., 2018). The addition of healthier natural ingredients, such as various rice powder substitutes for sugar, would therefore be expected to lead to the production of value-added gummy confections with associated health benefits (Jiamjariyatam, 2018).

**Table 4:** Physiochemical analysis of the gummy jelly samples containing the various added rice powders.

Rice powder	Composition (means ± SDs (*n* = 3))	
	Protein (%)	Carbohydrate (%)
Control	8.76 ± 0.43^a^	55.75 ± 0.51^c^
Thai jasmine rice	9.16 ± 1.51^a^	63.91 ± 0.43^b^
Thai white rice	8.93 ± 0.22^a^	64.43 ± 1.26^b^
Thai black sticky rice	10.01 ± 0.45^a^	69.52 ± 1.52^a^
Rice berry	9.73 ± 0.11^a^	63.24 ± 0.95^b^

Different lowercase letters within a column indicate significant differences (*P* ≤ 0.05).

Table 5 shows the L* values, which correspond to the lightness of the sample. As indicated, the L* values of the control (no added rice powder), Thai jasmine rice, Thai white rice, Thai black sticky rice, and rice berry rice were 31.14 ± 0.21, 34.27 ± 1.36, 31.01 ± 1.36, 33.75 ± 1.12, and 30.35 ± 1.31, respectively. In addition, the sample redness was determined according to the a* values, which were 2.42 ± 0.15, 2.57 ± 0.21, 2.26 ± 0.18, 2.71 ± 0.27, and 3.05 ± 0.22 for the control, Thai jasmine rice, Thai white rice, Thai black sticky rice, and rice berry rice samples, respectively. Furthermore, the yellowness of each samples was measured in terms of the b* value, giving values of 2.15 ± 0.56, 2.97 ± 0.66, 4.21 ± 0.44, 2.35 ± 0.47, and 1.86 ± 0.43 for the control, Thai jasmine rice, Thai white rice, Thai black sticky rice, and rice berry, respectively. Thai white rice had the lowest a* value and the highest b* value (*P* ≤ 0.05). Moreover, we note that Kongwan et al. (2017) used the response surface method to study the effects of various ingredients (i.e., GBRP, gelatine, and water) on the development of jelly products from GBRP, which resulted in L*, a*, and b* of 94.80, −0.14, and 1.57, respectively; the L* values of these jelly products were higher than those obtained in this study, with jasmine rice, Thai white rice, Thai black sticky rice, and rice berry giving values of 34.27 ± 1.36, 31.01 ± 1.36, 33.75 ± 1.12, and 30.35 ± 1.31, respectively. The ΔE* values of the samples represent the total color differences, which were 1.84 ± 1.42, 1.54 ± 1.02, 1.29 ± 0.72, 1.65 ± 1.09, and 2.09 ± 1.26 for the control, Thai jasmine rice, Thai white rice, Thai black sticky rice, and rice berry samples, respectively. These results indicate that the products were light in color, and the highest redness and yellowness values were found in rice berry rice and Thai white rice, respectively. The gel strengths of the control, Thai jasmine rice, Thai white rice, Thai black sticky rice, and rice berry samples were not significantly different (*P* > 0.05) from each other (Table 5) and were lower than those obtained by Tohmadlae et al. (2019) (i.e., 1,811.73 ± 8.80 g). In addition, their gelatine yields (i.e., 20.37 ± 0.64 %) from Nile tilapia skin were higher than those reported herein (17.33 %). It should be noted there that the stronger the gel network, the higher the gel strength, and so the discrepancy between our results and those obtained by Tohmadlae et al. (2019) may be attributed to the intrinsic molecular weight distribution and amino acid degradation, which affect the formation of peptides with shorter chain lengths, and reduce the ability of the resulting structures to form junction zones or undergo annealing (Kaewdang et al., 2015). According to our analysis of the pasting properties, the highest gel strength value was obtained for rice berry, and this was in accordance with the setback value of the rice berry powder.

**Table 5:** Analysis of the color and gel strength properties of the gummy jelly samples containing the various rice powders.

Group	Parameters (means ± SDs (*n* = 3))					
		L*	a*	b*	ΔE*	Gel strength (g)
Control		31.14 ± 0.21^b^	2.42 ± 0.15^bc^	2.15 ± 0.56^bc^	1.84 ± 1.42^a^	430.11 ± 52.38^a^
Thai jasmine rice		34.27 ± 1.36^a^	2.57 ± 0.21^bc^	2.97 ± 0.66^b^	1.54 ± 1.02^a^	489.82 ± 88.51^a^
Thai white rice		31.01 ± 1.36^b^	2.26 ± 0.18^c^	4.21 ± 0.44^a^	1.29 ± 0.72^a^	449.89 ± 78.80^a^
Thai black sticky rice		33.75 ± 1.12^a^	2.71 ± 0.27^ab^	2.35 ± 0.47^bc^	1.65 ± 1.09^a^	434.25 ± 68.50^a^
Rice berry		30.35 ± 1.31^b^	3.05 ± 0.22^a^	1.86 ± 0.43^c^	2.09 ± 1.26^a^	556.93 ± 33.26^a^

Different lowercase letters within a column indicate significant differences (*P* ≤ 0.05).

### Sensory evaluation

Table 6 presents the results of sensory evaluation, revealing insignificant differences among treatments (*P* > 0.05). However, the overall likeability score from the sensory panel for the proposed gummy jelly product with added rice powder was 7.13 ± 1.27. In terms of the “Like” to “Dislike” percentage responses, it received an “acceptable” rating; the nine-point hedonic scale indicated that the product was acceptable to 90.00 % of respondents. In addition, the gummy jelly with added rice berry had the highest sensorial score for the fishy odor parameter (6.97 ± 1.26), followed by the fishy flavor (6.93 ± 1.02), rice flavor (6.37 ± 0.75), sweetness (5.63 ± 0.93), and viscosity (6.63 ± 1.56) parameters, while Thai jasmine rice gave the highest score for color (6.90 ± 1.43).

**Table 6:** Sensory evaluation of the gummy jelly samples containing the various rice powders.

Attributes	Scores (means ± SDs (*n* = 50))			
	Thai jasmine rice	Thai white rice	Thai black sticky rice	Rice berry
Color	6.19 ± 1.48^a^	6.90 ± 1.43^a^	5.32 ± 1.46^a^	6.30 ± 0.54^a^
Fishy odor	6.23 ± 0.95^a^	6.57 ± 1.59^a^	6.39 ± 1.25^a^	6.97 ± 1.26^a^
Fishy flavor	6.35 ± 1.27^a^	6.43 ± 1.48^a^	6.29 ± 1.87^a^	6.93 ± 1.02^a^
Rice flavor	6.29 ± 1.52^a^	6.10 ± 0.76^a^	6.13 ± 0.91^a^	6.37 ± 0.75^a^
Sweetness	5.58 ± 1.34^a^	5.43 ± 0.71^a^	5.55 ± 0.96^a^	5.63 ± 0.93^a^
Viscosity	5.94 ± 0.86^a^	5.70 ± 1.55^a^	5.35 ± 1.23^a^	6.10 ± 1.65^a^
Overall liking	6.06 ± 0.96^a^	6.27 ± 0.45^a^	5.77 ± 1.44^a^	6.63 ± 0.88^a^

Different lowercase letters within a row indicate significant differences (*P* ≤ 0.05).

### Microbiological characteristics

Microbiological analysis revealed that the TPC, yeast, and mold contents of the proposed product were < 100 colonies per 1 mL of product. According to the Thai FDA guidelines for jams, jellies, and marmalades in hermetically sealed containers, standard regulation no. 213 (Thai FDA, 2000), there should be no contamination with pathogenic bacteria; however, a TPC of < 100 colonies in a 1 mL sample is acceptable. Moreover, there was no detectable microbial growth in any of the proposed gummy jelly products that contained the various varieties of added rice powder.

## Conclusion

The results of this study indicate that the tilapia skin as a waste product waste has the potential to be used in the food industry as a major source of gelatine, which can be successfully used as an ingredient for gummy jelly energy snacks containing added rice powder. Furthermore, based on several sensory parameters, the gummy jelly products created by combining this gelatine with rice powder were acceptable to a clear majority of test panelists. Physiochemical property analysis indicated that the proposed gummy jelly products with added black sticky rice powder had the highest protein and carbohydrate contents, while those containing Thai jasmine rice powder exhibited the highest brightness values, and rice berry powder resulted in the highest enjoyment value, with 90 % acceptance on a nine-point hedonic scale. In addition, microbiological analysis indicated that only trace contamination existed in the various samples, thereby confirming that the products were safe for consumption, as they conformed to the Thai Food and Drug Administration regulations. Based on these findings, gummy jelly prepared from tilapia gelatine and rice powder has the potential to be used as an energy snack, and due to their high protein and carbohydrate contents, the development of carbohydrate-protein supplements for athletes to consume during exercise can also be envisaged. Future research could target the development of new gummy jelly as a functional sports food for specific groups of athletes.

## CRediT authorship contribution statement

**Sukhuntha Osiriphun:** Conceptualization, Methodology, Software, Validation, Formal analysis, Investigation, Data curation, Writing – original draft, Writing – review & editing, Visualization, Supervision, Project administration, Funding acquisition. **Pornchai Rachtanapun:** Conceptualization, Validation, Formal analysis, Investigation, Data curation, Writing – original draft, Writing – review & editing, Supervision, Project administration. **Sutee Wangtueai:** Conceptualization, Methodology, Software, Validation, Formal analysis, Data curation, Writing – original draft, Writing – review & editing, Supervision, Project administration. **Wachira Jirarattanarangsri:** Conceptualization, Validation, Formal analysis, Data curation, Writing – original draft, Writing – review & editing, Supervision, Project administration.

## Declaration of Competing Interest

The authors declare that they have no known competing financial interests or personal relationships that could have appeared to influence the work reported in this paper.
